# Large language model integrations in cancer decision-making: a systematic review and meta-analysis

**DOI:** 10.1038/s41746-025-01824-7

**Published:** 2025-07-17

**Authors:** Yuexing Hao, Zhiwen Qiu, Jason Holmes, Corinna E. Löckenhoff, Wei Liu, Marzyeh Ghassemi, Saleh Kalantari

**Affiliations:** 1 Department of Radiation Oncology, Mayo Clinic, Phoenix, AZ USA; 2 Cornell University, Ithaca, NY USA; 3 Massachusetts Institute of Technology, Cambridge, MA USA

**Keywords:** Decision making, Cancer

## Abstract

Large Language Models (LLMs) are increasingly used to support cancer patients and clinicians in decision-making. This systematic review investigates how LLMs are integrated into oncology and evaluated by researchers. We conducted a comprehensive search across PubMed, Web of Science, Scopus, and the ACM Digital Library through May 2024, identifying 56 studies covering 15 cancer types. The meta-analysis results suggested that LLMs were commonly used to summarize, translate, and communicate clinical information, but performance varied: the average overall accuracy was 76.2%, with average diagnostic accuracy lower at 67.4%, revealing gaps in the clinical readiness of this technology. Most evaluations relied heavily on quantitative datasets and automated methods without human graders, emphasizing “accuracy” and “appropriateness” while rarely addressing “safety”, “harm”, or “clarity”. Current limitations for LLMs in cancer decision-making, such as limited domain knowledge and dependence on human oversight, demonstrate the need for open datasets and standardized evaluations to improve reliability.

## Introduction

Large language models (LLMs) are advanced AI systems that can parse inputs and generate human-like text responses to free-text queries, often with little or no task-specific fine-tuning^1^. These sophisticated models enable users to conversationally interact in a way that resembles engaging in a conversation with an authentic human interlocutor^2^. LLMs perform complex natural language processing (NLP) tasks by leveraging neural network architectures to identify associative relationships between words from large-scale datasets through a computationally intensive training process^3,4^. Recent LLM models, such as OpenAI’s ChatGPT, have demonstrated the ability to achieve high performance through prompt engineering, which involves the user’s ability to craft specific prompts to guide the model’s responses^5,6^. LLMs have shown promising results in generating acceptable responses to cognitive tasks across various fields, including medicine and healthcare^7–10^.

Applications of LLMs to a variety of clinical tasks in cancer care have rapidly expanded in recent years^11–15^. Successes include aiding decision-making in lung, breast, and prostate cancers, with uses in diagnosis, treatment recommendations, and patient education^16–18^. For instance, Peng and colleagues^19^ assessed ChatGPT’s efficacy in answering 131 colorectal cancer questions for diagnosis and treatment compared to clinical physicians. Their findings indicated that the LLM had high accuracy and comprehensiveness in domains such as radiation therapy and stoma care. Similarly, Yalamanchili and colleagues^20^ evaluated the effectiveness of LLMs in providing responses to 115 radiation oncology patient care questions using domain-specific expertise and domain-agnostic metrics. Their findings indicated that the LLMs’ responses were on par or superior to expert answers in 94% of cases for correctness, 77% for completeness, and 91% for conciseness. LLMs have also been applied to translate radiology reports for educating patients and to assist healthcare providers in communicating medical information. Examples include translating chest computed tomography lung cancer screening scans and prostate magnetic resonance imaging (MRI) reports, in both cases demonstrating high efficacy in correctness and completeness^21,22^.

Most existing reviews of related research literature focus on tangential areas, such as adopting non-LLM conversational AI agents in oncological care^5,23^, examining the technical aspects of LLMs in healthcare applications (i.e., model size and computational architecture)^12,24,25^ or reviewing LLM use in domain-specific areas such as skin cancer, urological cancer, or breast cancer management^11,26–30^. The limited focus and scope of these prior reviews leave a gap when it comes to synthesizing the overall state of the field for LLMs and cancer clinical decision-making. Despite the substantial clinical applications of LLMs in this area, we were unable to find any existing comprehensive assessment of their effectiveness, particularly in terms of real-world outcomes. The current systematic review and meta-analysis seek to address this gap by synthesizing previous research on the value of LLM chatbots for the cancer clinical decision-making process, patient health outcomes, and user experience.

To guide this critical synthesis, we center the systematic review around three focal areas: (1) the state of the art in LLMs across different cancer types in clinical decision-making; (2) the role of human factors, which is to say how clinical professionals and patients contribute to data generation and the evaluation of LLM outputs; and (3) identifying the datasets supporting cancer decision-making tasks and the evaluation approaches used across studies to assess LLM outcomes. By structuring the review around these dimensions, we aim to move beyond the narrower technical or disease-specific foci of previous reviews and provide a comparative overview of how LLMs are currently positioned within the cancer clinical decision-making process, as well as identify key challenges and outline steps needed to achieve clinical readiness.

## Results

### Study selection

**Fig. 1:**
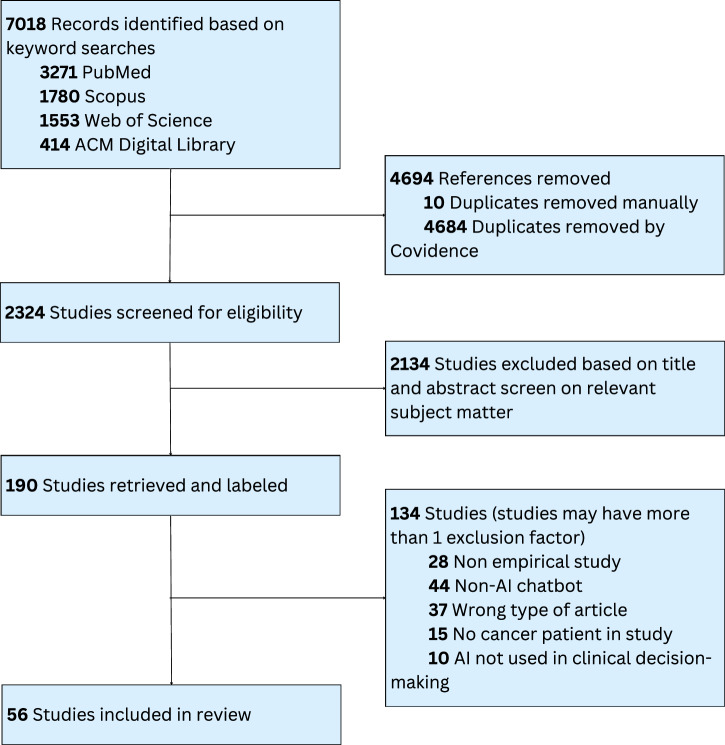
PRISMA flowchart showing the selection of studies.

We used the Covidence software platform (www.covidence.org) to automatically remove duplicate items from the total pool of 7018 articles. This left us with 2324 unique articles, which were then screened by their titles and abstracts according to our predefined inclusion criteria (inclusion and exclusion details are in the section “Search strategy”). During the abstract screening phase, most of the studies were excluded because they did not involve the use of AI-based chatbots in clinical decision support for cancer patients, and thus fell outside the scope of our review. Following the abstract screening, we were left with 190 articles, all of which were retrieved for a full-text eligibility evaluation. Additional studies were removed for failing to address cancer clinical decision-making, focusing on irrelevant technologies, or lacking sufficient data on patient outcomes. After this multistage screening process, 56 studies remained for inclusion in the review. A PRISMA flowchart outlining the article selection process is provided in Fig. 1.

### Study characteristics

Of the 56 included studies, 26 (46.4%) were published in 2023, while the remaining 30 (53.6%) were published in 2024. The lack of earlier studies is unsurprising, since LLMs only began to achieve widespread use around the year 2022, following the release of ChatGPT. Most of the studies were conducted by researchers in the United States (*n* = 21), followed by Germany (*n* = 6), China (*n* = 5), Turkey (*n* = 5), South Korea (*n* = 5), Canada (*n* = 2) and Denmark (*n* = 2). One study each was contributed by Brazil, Austria, Japan, Australia, Singapore, Argentina, India, the UK, Saudi Arabia, and Switzerland (for some papers that did not clearly state the study’s location, we used the institutional affiliation of the study’s first author to determine the associated country). A pie chart illustrating this geographical distribution of studies is provided in the Supplementary Fig. 1.

In terms of LLMs that were used, 55 (98. 2%) studies included OpenAI ChatGPT. More specifically, 33 (58.9%) included GPT-3.5, while 23 (41.1%) included GPT-4. Other LLMs that were assessed included Google Bard (*n* = 7, 12.5%), Bing Chat (*n* = 3, 5.4%), Claude (*n* = 1, 1.8%), YouChat (*n* = 1, 1.8%), and IBM Watson Assistant (*n* = 1, 1.8%). Some of the studies evaluated more than one LLM.

**Fig. 2:**
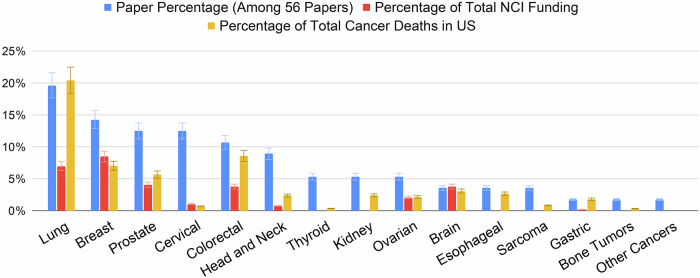
Types of cancer addressed in the reviewed studies. Each blue bar shows the proportion among the 56 papers, the red bar stands for the percentage of the specific cancer’s funding among the estimated total NCI budget funding in 2023^31^, orange bar stands for the percentage of the US cancer death rate in 2024^32^.

Fifteen types of cancer were represented in the studies, with lung cancer (*n* = 11), breast cancer (*n* = 8), prostate cancer (*n* = 7), and cervical cancer (*n* = 7) being the most frequently discussed (Fig. 2). To examine the relationship between research focus, funding, and cancer mortality, we compared the percentage of total National Cancer Institute (NCI) funding in 2023^31^ for each cancer type in this review with its projected share of total cancer deaths in the U.S. in 2024. Breast cancer received the highest NCI funding (8.5%, $580.6 million), followed by lung cancer (7.0%, $477.4 million) and prostate cancer (4.1%, $280.5 million). Funding data for thyroid cancer, kidney cancer, esophageal cancer, sarcoma, bone tumors, and other unspecified cancers were not available. In 2024, the American Cancer Society estimated 611,720 cancer-related deaths in the United States^32,33^. Lung cancer accounted for the highest proportion (20.5%), followed by colorectal cancer (8.65%) and breast cancer (7.1%). Mortality data for head and neck cancers, thyroid cancer, sarcoma, urological cancers, oropharyngeal cancer, bone tumors, and other specified cancers were not detailed in the projections. Supplementary Table 2 provides details for each cancer and the corresponding NCI budget funding and mortality rate. We observed an overall positive relationship between the number of papers focused on using LLMs in cancer education and decision-making vs the specific cancer’s mortality burden. (There were some exceptions, for example, cervical cancer is highly represented in the LLM literature, but has a relatively low mortality). This trend in the research literature often diverged from the NCI funding allocation; for example, lung cancer has the highest mortality burden and has received the most attention in LLM research, but its NCI funding is proportionally lower. Gaps in resource allocation for some of the most deadly cancer types, such as lung, colorectal, and (to a lesser extent) prostate, may negatively affect the ability of LLM researchers to conduct robust, large-scale studies, despite the clear interest in those cancers shown in the literature.

Several studies addressed general cancer treatments, such as radiotherapy and dermato-oncology, rather than specific cancer types, which are grouped under the “Other Cancers” category in the Fig. 2). Among the various aspects of cancer decision making, 22 studies (39.3%) examined treatment planning and recommendations, 18 (32.1%) examined general basic knowledge of cancer-relevant information and 17 (30.4%) investigated diagnosis from medical imaging or related questions. There were also 10 (17.9%) studies addressing prognosis and post-treatment management, 9 (16.1%) on screening and prevention, and 5 (8.9%) on report-summarization of patient profiles. Most of the studies addressed multiple decision-making aspects and were therefore included in multiple categories. More detailed numbers for the study domains are presented in the Supplementary Table 1.

As all studies included in this systematic review required human participants, 24 studies focused on patient-centered information, while 32 examined clinician-centered information. We classified studies as patient-centered if the primary end-users were patients, with some also including clinical professionals for evaluation. Clinician-centered studies were designed for clinician use and often relied on patient cases for development and validation. To compare participant composition, we defined “professionals” as clinicians, nurses, and clinical research coordinators, while “end-users” included cancer patients, survivors, and family caregivers. Among the 56 studies reviewed, 69.6% (*n* = 39) did not report the number of clinical professionals involved, and 19.6% (*n* = 11) did not specify the number of participating cancer patients. The absence of clear reporting on participant involvement limits the ability to assess the clinical relevance and generalizability of these findings.

**Fig. 3:**
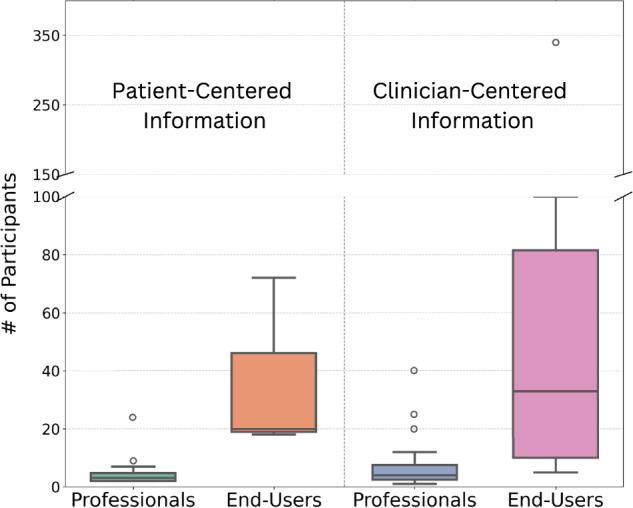
Number of clinical professional participants vs non-expert end user participants in studies of LLM performance.

On average, patient-centered studies included 4.55 professionals (SD = 4.73) and 36.67 end-users (SD = 30.62), whereas clinician-centered studies had larger participant pools, with 7.87 professionals (SD = 9.62) and 68.7 end-users (SD = 98.47). Clinician-centered studies generally involved more participants across both groups. Figure 3 presents a comparison of participant numbers in patient-centered and clinician-centered studies.

### Current trends in the dataset evaluation and human assessment of LLMs in cancer research

We categorized evaluation types based on the involvement of human judgment. In this analysis, “with human evaluations” refers to assessments involving interpretive methods such as qualitative interviews, open-ended responses, or narrative analysis to examine human responses to LLM output, such as appropriateness or comprehensiveness. In contrast, “automated evaluations” (or those without human input) refer to structured approaches such as using an LLM-as-a-grader, numerical scoring, or statistical analysis to assess textual outcomes like accuracy, reliability, or relevancy.

We also classified dataset types according to the nature of the data collected. “Qualitative data” refers to open-ended responses, narrative descriptions, or interview transcripts, which were typically analyzed through human-mediated thematic or interpretive methods. “Quantitative data” refers to structured numerical values such as scores, ranks, or frequency counts that can be subjected to statistical analysis. Some studies used mixed evaluation data, containing both qualitative and quantitative components.

**Fig. 4:**
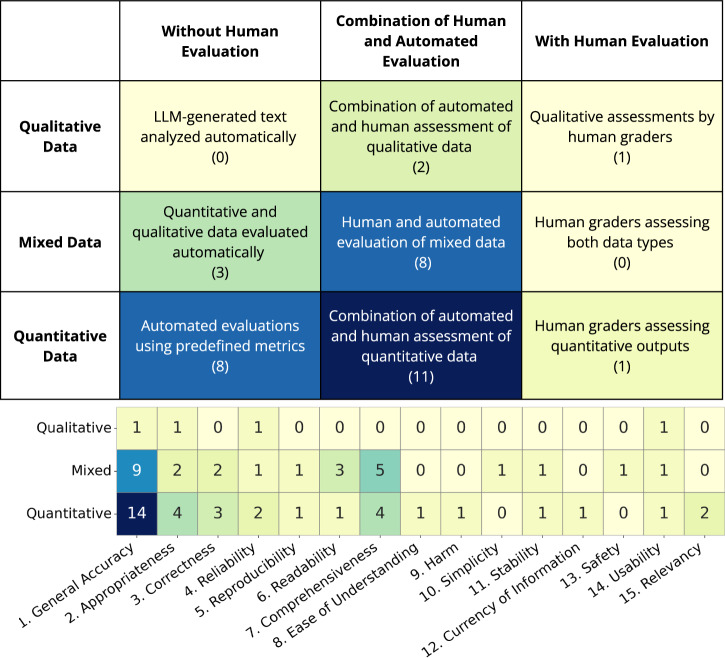
Heatmap overview of the evaluation metrics used in the reviewed studies. (Top) The table presents a 3 × 3 matrix defining the data and evaluation types, with parentheses indicating the number of papers in each category. Each paper is assigned to only one category. (Bottom) The heatmap illustrates the 15 evaluation categories identified across 34 salient papers, following the same data type classification as the top table. Unlike the top table, a single paper may employ multiple evaluation metrics and thus fall into multiple categories. The complete breakdown of data types and evaluation metrics for each paper is provided in Table 2.

Among the 34 papers that disclosed both evaluation datasets and evaluation metrics, we conducted a categorization analysis to identify methodological trends. Most studies were clustered in the categories that relied on automated evaluation and quantitative data (Fig. 4, at top). More than one-third of the studies (12 out of 34, 35.3%) did not include any human evaluation of the LLM output at all. There were only a small number of studies that relied solely on human evaluation methods; however, we found these studies to be particularly valuable and robust in the sense of capturing more nuanced insights about the successes and failures of the LLM-generated text. Such studies are vital to improve our understanding of complex factors such as user perceptions, satisfaction, trust, and comprehension, and for thoroughly analyzing interactions between clinicians and patients as mediated or influenced by LLM-generated text.

Across the evaluation metrics reported, we identified 15 specific evaluation constructs, with some overlapping categories (Fig. 4, at the bottom). The most commonly used outcome variables were “general accuracy,” “comprehensiveness,” and “appropriateness,” which were instrumentalized in various studies as qualitative or quantitative data. In the automated (without human) evaluations, additional focus was placed on “correctness,” “reliability,” and “relevancy,” whereas these aspects were rarely addressed in qualitative data. In contrast, the constructs of “simplicity” and “safety” appeared only in qualitative form (both in mixed-methods studies). The overall distribution of these evaluation constructs indicates that many important aspects of LLMs in cancer decision making are severely under-studied, with topics such as “safety,” “ease of understanding,” and “currency of information” (among others) appearing in only a very small number of studies. Full definitions of these evaluation categories and the corresponding scales used in the systematic review are presented in Supplementary Table 3.

The prevalence of quantitative evaluation approaches in this literature may not fully capture the complexity of deploying LLMs in the cancer domain. Clinical decision-making is not solely a technical process; it involves intricate human interactions and a great deal of complex assessment based on the details of an individual patient’s circumstances. We should expect the ways in which clinicians and patients interact with LLMs to be equally nuanced and complex. Studies that integrate both quantitative and qualitative evaluations of user experience are likely the best suited to identify gaps in current implementations and better optimize LLMs for real-world applicability. Such mixed-methods research can examine user trust, interpretability, and cognitive burden in greater depth, and attending to these human-centered factors is essential for assessing the clinical readiness of LLM-based tools.

### Meta-analysis

We conducted a meta-analysis for the datasets of 19 (33.9%) of the 56 studies. Those included in the meta-analysis were selected based on the following criteria: contained at least 20 questions or 10 cases to ensure sufficient statistical power, involved at least two experienced clinicians as graders to evaluate LLMs’ outputs, and provided extractable and standardizable data from the original article (allowing for calculations of correctness and quality of response).

**Fig. 5:**
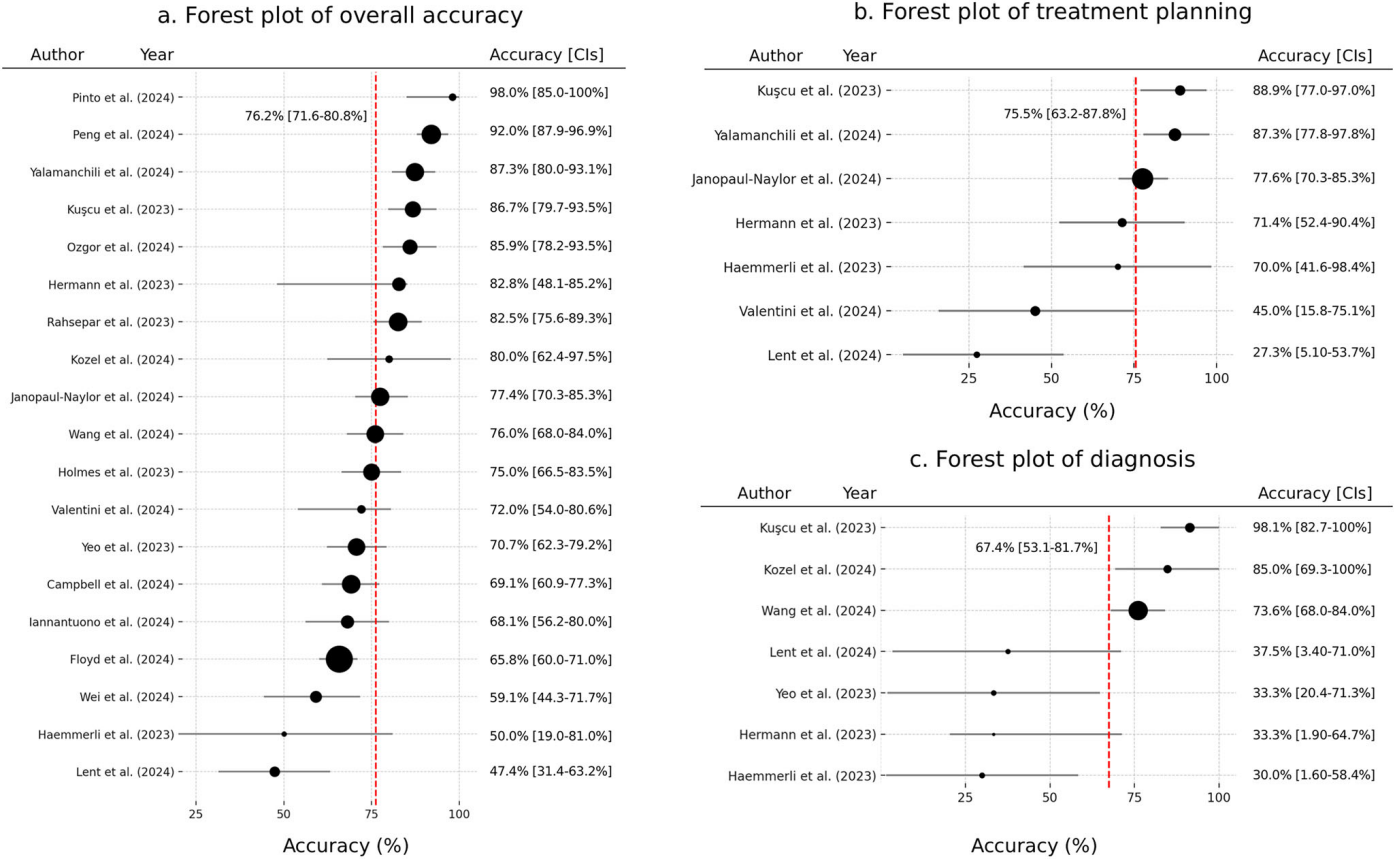
Forest plots showing meta-analyses of the reviewed literature. The figure presents LLM accuracy rates found across different study categories, with the circle sizes indicating sample size. **a** Shows findings for “general accuracy” of LLMs across all included meta-analysis studies, with sample sizes ranging from 20 to 131. **b** Focuses on “general accuracy” rates for studies that specifically examined cancer diagnosis, where the sample sizes ranged from 10 to 117. **c** Illustrates the “general accuracy” rates for studies centered on treatment planning, where the sample sizes ranged from 3 to 109. The red dotted lines show the mean values.

The results of the meta-analysis indicated that the overall average accuracy rating for LLM-assisted cancer decision making was 76.2% (95% CI: 71.6–80. 8%), with a standard error of 2.33% (Fig. 5a). Different studies’ outcomes showed moderately high heterogeneity in these ratings (*Q* = 53.8, *p* < 0.01, *I*^2^ = 67.2%), indicating that some studies rated LLM performance significantly higher than other studies. This heterogeneity may be attributed to variations in the study designs, as well as the risk of rating bias, the different LLM models used, and differences in evaluation metrics. For example, Kuşcu and colleagues^34^ employed a 4-point Likert scale to assess the accuracy of responses generated by ChatGPT for 154 head and neck cancer-related questions, whereas Peng and colleagues^19^ used a comprehensive scoring system on a 0–1 scale to evaluate completeness, correctness, and comprehensiveness for colorectal cancer-related questions. Such widely different methods could potentially lead to different conclusions about the LLMs’ performance.

To examine the impact of LLM outputs on decision making more closely and further differentiate this literature, we conducted subgroup analyses by examining the subgroups of studies focusing on treatment planning (*n* = 7) and those addressing diagnosis (*n* = 7). Regarding the use of LLMs in treatment planning, the meta-analysis found that the average reported quality and correctness rating was 75.5% (95% CI: 63.2–87.8%), with a standard error of 6.30% (Fig. 5b). This is roughly equivalent to the overall accuracy ratings (Fig. 5a). The analysis also indicated moderately high heterogeneity (*Q* = 72.3, *p* < 0.01, *I*^2^ = 92.7%) across the treatment-oriented studies.

Among those studies that evaluated LLMs for cancer diagnosis, the mean accuracy rating was a bit lower at 67.4% (95% CI: 53.1–81. 7%), with a standard error of 7.33% (Fig. 5c). Again the heterogeneity was significantly high (*Q* = 153.4, *p* < 0.01, *I*^2^ = 96.1%). In this case, the heterogeneity appeared to be strongly linked to differences in sample sizes among the included studies. For instance, Wang and colleagues^35^ reported a 73.6% accuracy for the GPT-4 model in relation to diagnosing 109 diverse thyroid cancer cases; while Haemmerli and colleagues^36^ reported a very poor accuracy rate of 30% when GPT-3.5 was tasked with classifying glioma types in 10 brain cancer cases.

Several methodological differences likely contributed to the high levels of heterogeneity that we observed in the literature. For instance, Yalamanchili and colleagues^20^ reported an unusually high correctness rating of 87.3% for 41 treatment planning questions in radiation oncology care, using a five-point scale ranging from “much worse” to “much better” compared to expert consensus. Valentini and colleagues^37^ reported a lower accuracy of 45% for 25 sarcoma-related questions, assessing factual correctness on a five-point Likert scale from “strongly disagree” to “strongly agree” regarding the presence of factual errors. Differences in LLM prompt design were also evident. Studies employing context-rich, clinically detailed prompts generally reported higher accuracy, while studies using generic prompts showed lower accuracy. These inconsistencies in scoring criteria and prompt construction complicate comparisons across studies and underscore the need for standardized evaluation frameworks when assessing LLM outputs in treatment planning.

Among the 18 studies that investigated LLMs’ responses to general cancer knowledge inquiries, 13 reported strong performance in factual correctness, completeness, and appropriateness (65.8–95.7%). For example, Zhou and colleagues^38^ examined GPT-4’s performance in disseminating gastric cancer knowledge and providing consultation recommendations, and found that it achieved 91.3% appropriateness and 95.7% consistency in a gastric cancer knowledge test. An additional three studies^39–41^ in this cohort qualitatively evaluated the readability of LLM-generated patient education materials, suggesting and finding that the LLM-generated text was roughly comparable to online search resources and outcomes. One study compared LLM outputs to human-generated literature, and found that the LLM materials were preferred by 48% of the participating head and neck surgeons^19^. The additional 2 studies in this area noted significant challenges with inaccurate and/or outdated information in the LLM outputs, such as incorrect grading of actinic keratosis and the omission of conventional Olsen grading in skin cancer information^42^.

For the ten studies on cancer prognosis and post-treatment management, six reported high correctness and comprehensiveness (ranging from 69.1% to 86.4%), while two qualitatively highlighted the potential of LLMs to promote self-management and improve health literacy among patients. Davis and colleagues^40^ specifically noted that the LLMs produced higher accuracy and concordance rates for oropharyngeal cancer post-treatment questions compared to those related to diagnosis and treatment. The remaining two studies also suggested the need for integrating more domain-specific knowledge to address misinformation and the lack of essential contexts in the LLM outputs.

There were nine studies focusing on screening and prevention. Eight of these reported high accuracy and appropriateness (64–95%) of the LLM responses in providing screening recommendations that aligned with clinicians or established guidelines, such as those of the American Cancer Society (ACS). However, one study found that LLMs performed poorly in predicting screening recommendations and showed inconsistent results, with an overall quality rating of 45%. In the 5 studies that examined report summarization of cancer MRI or pathological screening scans, LLMs' outputs had high quality scores (3.4–4.3 out of 5) and high accuracy in detecting relevant tumors and malignancies (87.7–94.17%). However, the LLMs were found to be less accurate in drawing medical conclusions on the basis of these findings.

Four studies examined the user-experience aspects of the LLMs. The outcomes of this research were broadly positive, with participants describing them as user-friendly and easy to use. Hao and colleagues^43^, for example, evaluated the effectiveness of a shared decision-making tool for older adult cancer patients that provided graphical presentations and lay-language explanations. Their findings indicated that both patients and clinicians valued the system’s potential to improve health literacy and engagement.

## Discussion

State-of-the-art LLMs, such as GPT-4o, have demonstrated remarkable capabilities in parsing natural language inputs and generating human-like responses^44^. They are not 100% accurate or effective at conveying information, but neither are clinicians. In the context of cancer care, the capabilities of LLMs offer several advantages. First, LLMs can synthesize large volumes of clinical guidelines, research papers, and patient-specific data to help provide evidence-based recommendations tailored to individual cases. This can significantly reduce the time that clinicians need to spend reviewing the literature and performing data analysis. Second, cancer treatment often involves the input of medical oncologists, surgical oncologists, radiation oncologists, and other specialists. LLMs can serve as a central knowledge hub, facilitating real-time collaboration and helping to promote consistency in treatment plans. Third, by generating simplified explanations of complex medical concepts, LLMs can help clinicians communicate treatment options more effectively to patients, thereby empowering patients to actively participate in shared decision-making.

The most popular foundational LLMs in use today were trained on unprecedented amounts of human-generated text, allowing them to produce convincing results in areas such as the summarization of established knowledge (historical, scientific, etc.) and standardized test responses including the United States Medical Licensing Examination (USMLE) and graduate record examinations (GREs). These domains are well-represented in the training data, allowing LLMs to provide generally accurate responses, though they are still far from perfect and sometimes hallucinate by providing convincing-sounding but false outputs. Despite their impressive capabilities, there are significant concerns regarding the ability of LLMs to handle specialized medical knowledge, which is not as widely and consistently represented in the training data compared to other topics. Certain medical specialties, particularly those that are less commonly discussed or documented in publicly available texts, may not be adequately represented during the model’s training process. Furthermore, such reduced amounts of reliable text may shift the percentage of high-quality information vs low-quality information in the training sets, making errors more likely. LLMs are also particularly prone to generating errors when addressing nuanced and novel situations. These issues raise a crucial concern: LLMs may be less reliable when generating output for specific medical domains, potentially limiting their effectiveness in clinical applications. The nuanced understanding required to identify and treat rare diseases, as well as information related to emerging medical treatments and highly specialized areas of surgery, might not be sufficiently captured in LLM outputs. The ability of an LLM to generate reliable outputs on a specific topic will not be known until it is actually tested for that topic.

In response to such concerns, a growing body of research has focused on assessing the performance of LLMs within particular medical fields^13,15^. Researchers are increasingly examining how well LLMs perform when tasked with medical decision-making, diagnosis, and treatment recommendations, especially in areas where specialized knowledge is crucial. As the results of our review have shown, the outcomes of such studies to date have been highly heterogeneous, indicating that further careful evaluation is needed. Robustly studying the proficiency of LLMs in domain-specific knowledge will be vital to determining when and if they can be successfully and ethically deployed in clinical settings.

Despite their potential, integrating LLMs into cancer decision support systems poses several challenges. Based on our systematic review, we categorized the key risks into seven areas: bias, lack of real-patient data, harm and safety monitoring, data privacy and ethical oversight, equity and representation, generalizability, and reproducibility. Table 1 summarizes these risks along with corresponding recommendations and example approaches and Table 2 presents detailed information for the 56 papers included in this systematic review.

**Table 1:** Recommendations for mitigating risks in LLM-based cancer decision-making

Risk category	Risk description	Recommendations and sample approaches
Automation bias	Clinicians or patients may over-trust LLM-generated outputs without critical review.	Integrate LLMs within accountable clinical decision support systems that require mandatory human verification before adoption of model suggestions.
Lack of real-patient data	Many LLM evaluations rely on synthetic, curated, or small sample datasets, limiting their relevance to real clinical practice.	Conduct data gathering involving direct interaction with patients and clinical professionals.
Harm and safety monitoring	There is limited systematic evaluation of potential harms, adverse events, or unintended consequences resulting from LLM-generated clinical recommendations.	Develop robust safety and harm evaluation metrics and implement them in both pre-deployment validation and post-deployment monitoring.
Data privacy and ethical oversight	Patient data may be exposed during model development, and many studies lack clear reporting on ethical review processes.	Strengthen ethical oversight by requiring IRB review where applicable and adopting privacy-preserving machine learning practices.
Equity and representation	Non-representative training data risks reinforcing healthcare disparities.	Mandate demographic reporting and dataset audits in evaluation studies.
Generalizability	Findings based on non-diverse datasets may not apply to broader clinical populations.	Validate LLM outputs across expansive and diverse patient populations before deployment.
Reproducibility	Limited sharing of datasets, LLM prompts, and evaluation protocols reduces transparency and prevents independent replication of LLM results.	Promote open access to datasets, model prompts, and evaluation benchmarks to strengthen reproducibility.

**Table 2:** Summary information for all of the included papers

First author	Year of publication	Country	Targeted condition(s)	Evaluator type	Data type	Evaluation type(s)	Aspect of decision-making	Prompt engineering?	IRB approval?
Rahsepar et al.^17^^†^	2023	USA	Lung cancer	Radiological scientist	Mixed	3	Patient-centered information	No	Not specified
Yeo et al.^53^^†^	2023	Korea	Cirrhosis and hepatocellular carcinoma (HCC)	Board-certified/eligible transplant hepatologist reviewers	Mixed	1, 5, 7	Patient-centered information	No	Not specified
Fink et al.^54^	2023	Germany	Lung cancer	Unspecified (existing dataset)	N/A	N/A	Clinician-centered information	No	Not specified
Rao et al.^55^	2023	USA	Breast cancer	Unspecified expert reviewers	Quantitative	2	Patient-centered information	Yes	Not specified
Coskun et al.^56^	2023	Turkey	Prostate cancer	Urologists	Mixed	1	Patient-centered information	No	Not specified
Hermann et al.^57^^†^	2023	USA	Cervical cancer	Attending gynecologic oncologists	Quantitative	3	Clinician-centered information	No	Not specified
Horiuchi et al.^58^	2024	Japan	Tumors in neuroradiology	Unspecified (existing dataset)	N/A	N/A	Clinician-centered information	No	Exempt
Nguyen et al.^59^	2023	USA	Four cancers: breast, ovarian, colorectal, and lung	Unspecified medical students	N/A	N/A	Clinician-centered information	Yes	Not specified
Lukac et al.^18^	2023	Germany	Breast cancer	Unspecified (existing dataset)	N/A	N/A	Clinician-centered information	No	Yes
Gebrael et al.^60^	2023	USA	Metastatic prostate cancer	Unspecified (existing dataset)	N/A	N/A	Clinician-centered information	No	Yes
Chung et al.^21^	2023	USA	Prostate cancer	Radiation oncologists	Quantitative	1, 3, 7, 8, 9	Clinician-centered information	No	Exempt
Kuşcu et al.^34^^†^	2023	Turkey	Head and neck cancer	Experienced head and neck surgeons	Qualitative	1	Patient-centered information	No	Exempt
Janopaul-Naylor et al.^61^^†^	2024	USA	Unspecified cancer	Oncologists with 5 to 10 years of clinical experience	Quantitative	1	Patient-centered information	No	Exempt
Haemmerli et al.^36^^†^	2023	Switzer-land	Brain glioma	Neuro-oncologists, radio-oncologists, radiologists, neurosurgeons, neuropathologists and neurologists	N/A	N/A	Clinician-centered information	No	Yes
Choi et al.^16^	2024	Korea	Kidney cancer	Urological oncologists specializing in kidney cancer	Mixed	1, 4	Patient-centered information	No	Yes
Wei et al.^62^^†^	2024	USA	Head and neck cancer	Unspecified reviewers	Mixed	1, 6, 7	Patient-centered information	No	Yes
Choo et al.^63^	2024	Korea	Colorectal cancer	Unspecified (existing dataset)	Quantitative	4	Clinician-centered information	No	Yes
Campbell et al.^39^^†^	2024	USA	Thyroid cancer	Unspecified graders	Quantitative	3	Patient-centered information	Yes	Exempt
Griewing et al.^64^	2023	Germany	Breast cancer	Unspecified (existing dataset)	Quantitative	1	Clinician-centered information	No	Not specified
Alanezi et al.^65^	2024	Saudi Arabia	Unspecified cancer	Unspecified (existing dataset)	N/A	N/A	Patient-centered information	No	Yes
Lyu et al.^22^	2023	USA	Lung cancer	Experienced radiologists	N/A	N/A	Clinician-centered information	Yes	Not specified
Benary et al.^66^	2023	Germany	Cancers with genetic alterations (lung cancer and others)	Unspecified (existing dataset)	N/A	N/A	Clinician-centered information	No	Exempt
Braithwaite et al.^67^	2024	USA	Breast cancer	Experts in the fields of general internal medicine, family medicine, geriatric medicine, population health, cancer control, and radiology	Mixed	2, 6	Patient-centered information	No	Not specified
Peng et al.^19^^†^	2024	China	Colorectal cancer	Unspecified evaluators and practitioners	Quantitative	1, 7	Patient-centered information	No	Yes
Köroğlu et al.^68^	2023	Turkey	Thyroid cancer	Endocrinologists	Mixed	1, 13, 14	Clinician-centered information	No	Exempt
Ozgor et al.^69^^†^	2024	Turkey	Urological cancers: prostate, bladder, kidney, and testicular cancers	Experienced physicians in uro-oncology	Qualitative	14	Patient-centered information	No	Not specified
Davis et al.^40^	2024	USA	Oropharyngeal cancer	Unspecified physician-graders	Mixed	1, 7	Patient-centered information	No	Exempt
Son et al.^70^	2023	Korea	Breast, prostate, and lung cancer	Unspecified (existing dataset)	N/A	N/A	Clinician-centered information	No	Yes
Lee et al.^71^	2023	USA	Head and neck cancer	Experienced head and neck surgeons	N/A	N/A	Patient-centered information	No	Exempt
Arasteh et al.^72^	2024	Germany	Esophageal cancer	Unspecified (existing dataset)	N/A	N/A	Clinician-centered information	Yes	Yes
Pinto et al.^73^^†^	2024	Brazil	Prostate cancer	Urologists	Quantitative	1	Clinician-centered information	Yes	Exempt
Valentini et al.^37^^†^	2024	Austria	Sarcoma, a rare malignant tumor	Sarcoma experts (orthopedic oncologists and medical oncologists)	Mixed	1, 2, 7	Patient-centered information	No	Exempt
Thia et al.^41^	2024	Australia	Urological malignancies	Urology specialists	N/A	N/A	Patient-centered information	No	Exempt
Atarere et al.^74^	2024	USA	Colorectal cancer	Board-certified Internal Medicine physicians (internists) and an oncologic Gastroenterologist with significant colorectal cancer experience	Qualitative	2, 4	Patient-centered information	No	Not specified
Lim et al.^75^	2024	Singapore	Colorectal cancer	Unspecified (existing dataset)	Quantitative	1, 2	Clinician-centered information	Yes	Not specified
Choi et al.^76^	2023	Korea	Breast cancer	Breast cancer patients	N/A	N/A	Clinician-centered information	Yes	Yes
Wu et al.^77^	2024	China	Thyroid cancer	Unspecified reader	N/A	N/A	Clinician-centered information	No	Yes
Pereyra et al.^78^	2024	Argentina	Colorectal cancer	Board-certified gastroenterologists with more than 10 years of clinical experience	Quantitative	1	Clinician-centered information	No	Yes
Iannantuono et al.^12^^†^	2024	USA	Unspecified cancer	Unspecified expert reviewers	Quantitative	1, 4, 5, 6, 15	Clinician-centered information	No	Not specified
Chow et al.^79^	2023	Canada	Unspecified cancers in radiotherapy	Unspecified participants	N/A	N/A	Patient-centered information	Yes	Exempt
Yalamanchili et al.^20^^†^	2024	USA	Unspecified cancer	Radiation oncologists and physicists	N/A	N/A	Patient-centered information	Yes	Exempt
Kozel et al.^80^^†^	2024	USA	Brain tumors	Neurosurgeons	Quantitative	1	Clinician-centered information	No	Not required
Wang et al.^35^^†^	2024	China	Thyroid cancer	Doctors	N/A	N/A	Clinician-centered information	No	Yes
Yang et al.^81^	2024	China	Bone tumors	Experienced physicians (intermediate and senior doctors)	N/A	N/A	Clinician-centered information	No	Exempt
Hasani et al.^82^	2023	USA	Unspecified cancers in radiology	Urology fellows	N/A	N/A	Clinician-centered information	Yes	Yes
Zhou et al.^38^	2023	China	Gastric cancer	Unspecified graders	Quantitative	2, 11	Clinician-centered information	Yes	Not specified
Cocci et al.^83^	2023	Denmark	Urological related cancers	Urologist	N/A	N/A	Clinician-centered information	No	Not specified
Yucel et al.^84^	2024	Turkey	Unspecified cancer	Medical oncologists	Mixed	1, 6, 11	Patient-centered information	Yes	Not specified
Huo et al.^85^	2024	Canada	Colorectal cancer	Patients	Quantitative	1	Patient-centered information	Yes	Not specified
Sarangi et al.^86^	2023	India	Unspecified radiological cancers	Radiologists	Mixed	1, 3, 7, 10	Clinician-centered information	Yes	Not specified
Lent et al.^42^^†^	2024	Denmark	Dermato-oncology	Dermatologists	Quantitative	1, 7, 12, and 14	Clinician-centered information	Yes	Not specified
Floyd et al.^87^^†^	2024	USA	Unspecified cancers	Radiation oncology resident physicians	Quantitative	1, 7	Patient-centered information	No	Not specified
Gabriel et al.^88^	2023	UK	Cancer treatments	Urologists	Quantitative	1, 2, 15	Clinician-centered information	No	Not specified
Sievert et al.^89^	2024	Germany	Oropharyngeal squamous cell carcinoma	Surgeons and pathologists graders	N/A	N/A	Clinician-centered information	Yes	Yes
Hao et al.^43^	2024	USA	Unspecified cancer	Older adult cancer patient participants and cancer-related clinician participants	N/A	N/A	Patient-centered information	No	Yes
Holmes et al.^13^^†^	2023	USA	Unspecified cancer	Medical physics residents and research fellows, as well as non-physicians with expertise in radiation physics	Quantitative	1	Clinician-centered information	Yes	Not specified

Included papers and associated information, including publication year, first author’s country, target condition(s) of the paper, participant/grader roles, number of clinical professionals and non-expert participants, the decision-making aspect of the research, and whether the study involves prompt engineering. Fifteen types of evaluation metrics were identified in the reviewed literature; each was assigned a category number as follows: (1) general accuracy, (2) appropriateness, (3) correctness, (4) reliability, (5) reproducibility, (6) readability, (7) comprehensiveness, (8) ease of understanding, (9) harm, (10) simplicity, (11) stability, (12) currency of information, (13) safety, (14) usability, and (15) relevancy. Summary statistics and visualizations for the evaluation metrics are presented in Fig. 4. Detailed definitions of each metric and the corresponding scales referenced in the papers included in this systematic review are presented in Supplementary Table 3.

^†^Represents the papers included in the meta-analysis.

Errors or biases in the models’ outputs could lead to suboptimal or even harmful clinical decisions. LLMs trained on unbalanced datasets can perpetuate or amplify biases, affecting treatment recommendations for underrepresented populations. Ensuring fairness and inclusivity in the training data is essential, and current LLMs have shown significant failures in this area. Addressing these shortcomings will require not only technical interventions but also broader global representation in both datasets and research contexts, particularly from low-resource settings and historically marginalized populations. The adaptation of LLMs to oncology-specific use cases by incorporating domain-specific data and clinicians’ feedback can potentially mitigate such issues, as well as improve their overall accuracy. The text-generation processes used by LLMs are often opaque, and this lack of transparency can further hinder clinicians’ ability and willingness to trust their outputs when patients’ lives are on the line. In addition, cancer care involves sensitive patient data, and the use of LLMs raises concerns about data confidentiality. Ensuring LLMs’ compliance with regulations such as the Health Insurance Portability and Accountability Act (HIPAA) is a pressing concern. To guide the field toward more inclusive and contextually grounded evaluation standards, future work should adopt mixed-methods research designs that integrate quantitative performance metrics with qualitative insights from both clinical experts and patients.

The use of LLMs in cancer decision-making research is costly and time-consuming, especially since state-of-the-art LLMs require significant computing resources. This limits their use in low-resource settings. Furthermore, evaluating these technologies involves recruiting clinical professionals and patients, which may be more difficult in resource-constrained practice environments. In our systematic review, 37.5% of the studies came from the United States, 10.7% from Germany, and 8.9% from China. All together, the research was largely concentrated in a few high-income countries, and global representation was limited. Many of the studies did not report the demographic features of the patient and physician participants, which raises concerns about the generalization of their findings to diverse healthcare systems and patient groups.

Our review also identifies a crucial gap in current evaluation practices when it comes to safety and harm-related outcomes (for example, the possibility that LLM output might overlook vital safety cautions in its recommendations or fail to take into account drug interactions). Only two of the included studies explicitly examined these dimensions. Developing standardized evaluation frameworks that address the topic of patient safety is vital to ensure the responsible use of LLMs in cancer care and promote their adoption.

Beyond technical and logistical barriers, the integration of LLMs in cancer decision-making raises ethical concerns. Of the 56 papers analyzed, only 34 disclosed both their data and evaluation metrics. Our meta-analysis of 19 studies revealed low average accuracy rates, with an overall accuracy of 76.2% (treatment planning accuracy of 75.5%, and diagnostic accuracy of 67.4%). Given these relatively low performance levels, it is not feasible to automate LLM-driven predictions for diagnosis, treatment, or broader decision-making tasks in real-world oncology. There is a risk that financial pressures or a desire for “efficiency” could result in a rush toward automation before the safety and effectiveness of the technology has been robustly demonstrated, or that time-strapped clinicians may come to defer automatically to LLM outputs without giving them the necessary scrutiny. At the current time, expert oversight remains essential, and LLMs are far from ready to replace clinicians’ final judgment. At best, they may offer limited support in clinical decision-making or help automate simple clinical tasks prior to expert review^45^. While LLMs show potential in enhancing decision-making, they should not replace clinical professionals’ judgment. There is a danger that time-strapped clinicians may come to defer automatically to LLM outputs without giving them the necessary expert scrutiny. Striking the right balance between automation and clinicians’ oversight is crucial to maintaining accountability and patient trust. In addition, patients should be informed about the role of AI in their care and have the opportunity to consent to its use, without pressure or financial incentives. Transparent communication about the capabilities and limitations of LLMs is essential for all parties.

It is notable that in research evaluating LLM outcomes, our systematic review found a widespread reliance on automated evaluations and synthetic data, often neglecting more granular human oversights, despite our inclusion criterion that studies must involve cancer patients and/or clinical professionals. The evaluation methods were largely limited to Likert scales (*n* = 26) and true/false assessments (*n* = 13), approaches that are insufficient for fully capturing the complexity of cancer decision-making tasks. Most datasets focused narrowly on multiple-choice or short-answer question formats, which do not reflect the broader range of clinical decision-making processes. Successful integration of LLMs requires more than favorable statistical outcomes; it demands attention to interaction quality, usability, and interpretability within clinical workflows. Another concern is that 26.8% of the studies did not report the status of their Institutional Review Board (IRB) approval, exemption, or waiver. In clinical research, especially when dealing with sensitive patient data or interactions with vulnerable groups, failure to disclose IRB status raises serious ethical questions about how risks to participants were addressed. The lack of transparency not only limits the trustworthiness of the research but also suggests that ethical safeguards may have been overlooked. Many studies did not report participant demographics, and some did not even report the number of participants. This omission is not trivial. For LLMs to be responsibly integrated into cancer care, their impact on communication, understanding, emotional well-being, and shared decision-making must be carefully examined. Studies that treat accuracy as the primary endpoint miss the broader picture of what matters to patients in clinical settings.

The field would benefit from a shift toward richer, more robust, mixed-methods studies that capture user trust, interpretability, and cognitive load in more detail. Designing user-centered interfaces, embedding LLMs thoughtfully into clinical processes, and involving patients and healthcare professionals throughout LLM development will be essential. Feasibility studies conducted across diverse healthcare systems are also important to avoid reinforcing existing disparities. Greater transparency in how LLMs generate and present information can help in establishing their credibility and ensuring their responsible use in clinical care.

In the few months since we completed our literature search (on May 15th, 2024), several new studies have been published on salient topics^46,47^, which are not included in the current analysis. Given the rapid pace of advancements in this field, we can expect that even more research will be available prior to our publication^48,49^. It is therefore crucial to keep the state of review current by periodically reassessing the contours of the literature and tracking emerging trends. Such updates should take into account the evolving landscape of LLM technologies, as well as their evolving applications in oncology.

While the studies reviewed in this report generally demonstrated robust methods, there are improvements that could be made in future efforts to strengthen the quality of the research literature. The adequacy of validation and testing-set sizes in many of the studies could be improved to support greater generalizability of the findings. Ensuring that LLMs are evaluated across diverse patient populations and different clinical settings is essential to confirm their applicability in real-world healthcare settings. This concern extends to the LLM-based intervention design, which could benefit from greater stakeholder involvement, including inputs from diverse clinicians, patients, and advocates. Engaging a broad range of stakeholders is vital to ensure that LLMs are designed and evaluated in an equitable fashion.

Additional methodological concerns in the literature are persistent challenges related to bias in AI models. Many LLMs are trained on large datasets that may contain biased or unrepresentative data, which can result in unfair or inaccurate recommendations, particularly when addressing the personalized needs of individual patients. Addressing bias in the development and deployment of LLMs is a crucial part of ensuring that they can be used ethically for diverse patient populations. Finally, the practical implementation of LLMs in real-world medical contexts presents additional barriers. These include issues related to integration with existing healthcare workflows, clinician trust in AI-generated recommendations, and patient acceptance of AI-assisted decision-making. Improving the technical performance of LLMs may help in overcoming these challenges, but it will also be necessary to address human psychological and social needs when seeking to ethically integrate LLMs into clinical decision-making.

In this systematic review, we analyzed 56 papers focusing on the use of LLM-based applications in cancer clinical decision-making. The findings highlighted the potential of LLMs to enhance healthcare efficiency and communication, although concerns remain with the uneven accuracy of their outputs and potential for unethical use. The reviewed studies exhibited a great deal of heterogeneity in their findings, indicating a need for further robust research to assess LLM-based tools, particularly in highly specialized and newly emerging areas of medical expertise. To maximize the effectiveness of LLMs in healthcare applications, it will be essential to engage a diverse range of stakeholders, including clinicians, patients, and advocates, during the tools’ development and evaluation processes. Such engagement can help address crucial challenges to adoption, such as a lack of transparency in how LLMs generate responses and difficulties in interface usage. Furthermore, the quality of the LLM studies requires improvement, particularly by developing robust methodologies for assessing real-world impacts such as long-term clinical outcomes and patient satisfaction. Establishing standardized evaluation frameworks that integrate both subjective and objective measures will be key to generating actionable insights and determining what role LLMs may be able to play in cancer care.

## Methods

### Search strategy

The search of published academic studies was carried out in March of 2024, and the review protocol was registered with Open Science Framework (OSF)^50^ (DOI 10.17605/OSF.IO/Y983Q) and PROSPERO (CRD42024567756) in the same month^2^. To ensure a comprehensive evaluation of a diverse range of published articles, we selected four databases: PubMed, Scopus, Web of Science, and the ACM Digital Library. Scopus and Web of Science cover a broad spectrum of scientific literature, while PubMed focuses specifically on health-related topics, and ACM specializes in technology-related research. By combining these domain-specific databases with general scientific publications, we created a robust literature database highly suited to the goals of our project.

The following terms were used to search the titles, abstracts, keywords, and subject headings of articles: (“Chatbot” OR “Conversational Agent” OR “Conversational Artificial Intelligence” OR “Conversational AI” OR “Conversational Interface” OR “Large Language Model” OR “LLM” OR “ChatGPT” OR “Virtual Agent” OR “Digital Assistant” OR “Intelligent Assistant” OR “AI Assistant” OR “AI-powered Chatbot” OR “Voice Assistant”) AND (“Cancer” OR “Oncolog*” OR “Leukemia” OR “Lymphoma” OR “Sarcoma” OR “Carcinoma” OR “Radiation” OR “Radiotherapy” OR “Radiology” OR “Tumor” OR “Neoplasm” OR “Chemotherapy” OR “Malignancy” OR “Myeloma”) AND (“Clinical Decision Support” OR “Clinical Support System” OR “Clinical Decision-Making” OR “Clinical Guid*” OR “Diagnosis” OR “Predict” OR “Treatment” OR “Therapy” OR “Risk Assess*” OR “Screen” OR “Prognosis” OR “Patient Management” OR “Medical Imaging” OR “Electronic Health Records Decision support” OR “Non-knowledge-based Systems” OR “Therapeutic Monitoring” OR “Clinical Management” OR “Information Retrieval Tools” OR “Health Information Exchange” OR “Consultation” OR “Counseling” OR “Reassess*” OR “Rehabilitation” OR “Physical Examination” OR “Emotional Support” OR “Palliative Care”).

This string was intended to cover variations of the relevant words and closely related concepts that are often used interchangeably (e.g., chatbot vs conversational agent), while still keeping the search narrow enough to exclude the much broader literature on virtual reality. We consulted with an evidence synthesis librarian at our university to carefully formulate and apply this search string so that the review would achieve an effective scope. The results included 3271 items from PubMed, 1780 items from Scopus, 1553 items from Web of Science, and 414 items from the ACM Digital Library.

### Eligibility criteria

To ensure relevance, rigor, and coherence in the data compilation, we established clear inclusion and exclusion criteria. An item was required to satisfy all of the following conditions for inclusion: (1) the article is published in a peer-reviewed journal or conference proceeding. (2) The study presented in the article is based on empirical research involving cancer patients, utilizing qualitative and/or quantitative methods. For this review, a cancer patient is defined as an individual diagnosed with any type of cancer, who is awaiting or undergoing medical care and treatment. (3) The article discusses the use of LLM chatbots for cancer clinical decision-making support. (4) The study involves the use of the LLM-based technologies. (5) The paper is published in English.

Items were excluded from the review if they met any of the following conditions: (1) the paper is a literature review (including scoping, systematic, meta-analysis, or integrative reviews), dissertation, protocol, book, book chapter, overview article, workshop, poster, case study, or editorial (including perspectives, brief communications, correspondence, letters, editorials, or responses). (2) The intervention described is mainly based on non-AI methods, including knowledge-based systems, rule-based systems, scripted responses, keyword matching, template-based responses, and menu-based navigation. (3) The intervention is designed for applications other than cancer clinical decision-making; for example, LLM applications focused on providing mental health support for patients (defined as promoting psychological well-being or managing mental health conditions unrelated to cancer) or administrative tasks (defined as nonclinical activities including scheduling, billing, or care coordination) were excluded. (4) Empirical studies are excluded if the studied population primarily consists of non-patient demographics rather than medical patients.

### Screening process

Study selection was conducted using the Covidence systematic review management platform, beginning with an initial pool of 7018 articles (3271 from PubMed, 1780 from Scopus, 1553 from Web of Science, and 414 from the ACM Digital Library). A total of 4694 duplicate records were removed (4684 by the Covidence platform and 10 manually by two screeners), resulting in 2324 unique articles for screening. Two screeners independently screened the titles and abstracts of all articles in accordance with the predefined inclusion and exclusion criteria (see section “Search strategy”) and then compared results. During this phase, 2134 articles were excluded for clearly failing to meet the review’s inclusion/exclusion criteria, primarily due to not involving the use of AI-based chatbots in clinical decision support for cancer patients. At this stage, 134 articles were excluded: 28 were non-empirical studies, 44 did not involve AI-based chatbots, 37 were of the wrong publication type (e.g., literature reviews, workshops, editorials, or posters), 15 did not include cancer patients, and 10 did not involve AI or LLM in clinical decision making. Following this multistage screening process, 56 studies were retained for inclusion in the final review.

### Data extraction and analysis

We created a standardized form for summarizing the articles’ contents, and then divided the 56 articles among two researchers (Hao and Qiu) for review. In addition to recording the year of publication, authors’ names, type of publication, and the name of the publication outlet, we summarized each study’s content in the following categories: (a) research questions and hypotheses; (b) country in which the study took place; (c) type of LLM technology used; (d) types of cancer addressed; (e) types of clinical decisions addressed (diagnosis, treatment planning, etc.); (f) experiment design; (g) independent, dependent, and mediating variables; (h) number of participants and demographic breakdowns (including age and any specific medical conditions studied); (i) summary of results; (j) limitations and recommendations; and (k) other pertinent information. For most of these categories, we established predefined codes that could be readily applied, while remaining open to studies that might not fit within our a priori schema. For example, under the category of experiment design, we included codes such as between-subjects, within-subjects, or factorial design. Similarly, to understand the studies’ results, we identified whether the studies were oriented towards evaluating technological advancements, user experiences, or efficacy in cancer clinical decision-making. In addition to applying these codes, we noted the particulars of the results and any notable findings. After summarizing all of the studies in this fashion, we carefully reviewed and discussed the collected literature to identify emerging areas of consensus and contradiction.

### Study quality appraisal

The mixed methods appraisal tool (MMAT) was used to assess the quality of each included article^51^. We determined that 13 articles employed quantitative methods, one used a qualitative method, and the remaining 42 adopted a mixed-methods approach. Two screeners independently reviewed each article using MMAT and then discussed the results to attain final scores, following the process described by Evangelio and colleagues^52^. The average MMAT score for the quantitative studies was 5.4 on a 0–7 scale, with individual study scores ranging from 4.0 to 6.0. The single qualitative study scored 6.0 on a 0–7 scale. For the mixed-methods studies, the average score was 11.1 on a 0–17 scale, with individual study scores ranging from 5.5 to 14.0. A more detailed description of this scoring process and its results is provided in the Supplementary Data.

## Supplementary information

Supplementary Information

PRISMA_2020_checklist

Supplementary_Data

## Data Availability

All data underlying this study’s results, including the raw datasets used for the meta-analysis, are provided in the Supplementary Data. That same repository also houses the question-answering (QA) datasets and patient cases from the 33 reviewed studies that made their evaluation data publicly available, as well as the systematic review materials (evaluation datasets and the accompanying MMAT appraisal file).

## Code availability

No code was used for data processing or analysis.
