# Supplementary material for: Large language model integrations in cancer decision-making: a systematic review and meta-analysis
Source: NPJ Digit Med. 2025 Jul 17;8:450. doi: 10.1038/s41746-025-01824-7 (PMC12271406; doi:10.1038/s41746-025-01824-7)
Supplement: Supplementary file 1 — Supplementary Information [file 41746_2025_1824_MOESM1_ESM.pdf]

Supplementary Information

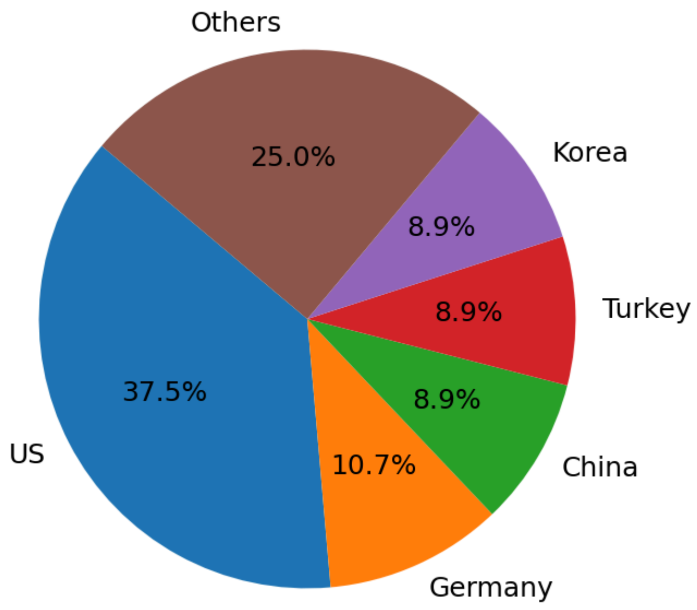

Supplementary Figure 1. Distribution of studies by country (based on the first author's location when country is unspecified)**.**

Supplementary Table 1. Summative Results per Category

Category	Number of Studies	Percentage (out of 56 papers)
**Years of Publication**		
2023	26	46.4%
2024	30	53.6%
**Types of Publication**		
Medical/Clinical-Related Domain	49	87.5%
Computer Science-Related Domain	7	12.5%
**Obtain IRB**		
Yes	18	32.1%
Exempt	15	37.5%
No	2	3.6%
Not Mentioned	21	26.8%
**Data Availability**		

Yes	37	66.1%
Questions Only	23	41.1%
Cases Only	11	19.6%
Questions & Cases	3	5.4%
No	19	33.9%
**Type of Study**		
Quantitative	43	76.8%
Qualitative	2	3.6%
Mixed Methods	11	19.6%
**Types of LLM Usage ***		
GPT (3.5 or/and 4)	55	98.2%
Google Bard/Gemini	7	12.5%
Bing Chat	3	5.4%
Claude	1	1.8%

You Chat	1	1.8%
IBM Watson Assistant **	1	1.8%
**Study Target Populations**		
Clinical Professionals	32	57.1%
Patient	24	42.9%
**Number of Studies Reporting Participant Numbers**		
Expert Professionals (including clinicians, nurses, or other clinical professionals)	48	85.7%
Non-Expert Participants (including patients, survivors, family caregivers)	17	30.4%

* Some papers used multiple LLMs to evaluate and assess the performance.

** IBM Watson Assistant did not employ transformer-based LLM architecture but rather operated as a rule-based system focused on intent classification and AI-based dialogue management. We considered it consistent with our inclusion criteria given its use of machine-learning-based conversational methodologies that align with the evolutionary trajectory of LLMs in clinical decision support contexts.

Supplementary Table 2. Summary of Cancer Types in the Review Papers, with NCI Funding and Mortality Estimates. A dash (–) indicates unknown or unreported values.

Specific Cancer Type	Paper Counts	Paper % (Among 56 Papers)	NCI 2023 Funding (Million in USD)	Funding %	Estimated 2024 New Cases	Estimated Deaths	Percentage of Total Cancer Death %
Lung	11	19.64%	$477.0	7.00%	234,580	125,070	20.45%
Breast	8	14.29%	$581.0	8.50%	297,790	43,170	7.06%
Prostate	7	12.50%	$280.5	4.10%	288,300	34,700	5.67%
Cervical	7	12.50%	$76.6	1.10%	14,100	4,280	0.70%
Colorectal	6	10.71%	$257.9	3.80%	153,020	52,550	8.59%
Head and Neck	5	8.93%	$55.1	0.80%	66,920	14,620	2.39%
Thyroid	3	5.36%	--	--	43,800	2,230	0.36%
Kidney	3	5.36%	--	--	81,800	14,890	2.43%
Ovarian	3	5.36%	$140.1	2.10%	19,710	13,270	2.17%
Brain	2	3.57%	$261.1	3.80%	25,280	18,990	3.10%
Esophageal	2	3.57%	--	--	20,640	16,410	2.68%
Sarcoma	2	3.57%	--	--	13,190	5,130	0.84%
Gastric	1	1.79%	$16.0	0.20%	26,500	11,130	1.82%
Bone Tumors	1	1.79%	--	--	3,970	2,140	0.35%
Other Cancers	1	1.79%	--	--	--	--	--

Supplementary Table 3. Evaluation Constructs and Associated Assessment Methods

Construct	Definition	Scales Found in Systematic Review
**General Accuracy**	The proportion of outputs that correctly answer the clinical question based on a predefined reference standard.	Likert scale, F1-score, expert binary grading (true/false)
**Appropriateness**	The degree to which responses are clinically suitable and aligned with accepted standards of care.	Likert scale, expert panel consensus
**Correctness**	The factual accuracy of content produced, regardless of clinical applicability.	Likert scale, Expert binary grading (correct/incorrect), proportion correct, sensitivity, specificity, and AUC
**Reliability**	The consistency of outputs across similar inputs and clinical scenarios.	Likert scale
**Reproducibility**	The ability to produce consistent outputs when identical inputs are provided under the same conditions.	Expert binary grading (true/false)
**Readability**	The extent to which outputs are written in clear, accessible language appropriate for clinical use.	Likert scale, Flesch-Kincaid Grade Level (FKGL), Flesch Reading Ease (FRE)
**Comprehensiveness**	The extent to which responses address all relevant aspects of the clinical question without omission.	Likert scale
**Ease of Understanding**	The subjective clarity with which users can interpret and act upon the model's outputs.	Likert scale
**Harm**	The potential for outputs to contribute to clinical errors or adverse patient outcomes.	Likert scale
**Simplicity**	The presentation of information in a concise and straightforward manner without unnecessary complexity.	Likert scale
**Stability**	The robustness of outputs when input phrasing or contextual details vary slightly.	Likert scale

**Currency of Information**	The extent to which outputs reflect current clinical evidence, guidelines, and standards.	Expert binary grading (yes/no)
**Safety**	The minimization of risk to patients based on the content and recommendations generated.	Likert scale
**Usability**	The extent to which outputs can be effectively and efficiently integrated into clinical workflows, including dimensions such as efficiency, dependability, stimulation, and novelty.	Likert scale, global quality score (GQS), user experience questionnaire (UEQ)
**Relevancy**	The alignment of outputs with the clinical question posed, avoiding irrelevant or extraneous information.	Likert scale

Supplementary Table 4. Meta Analysis Result Table.

Title	Diagnosis	Treatment	Combined	Reading	Corrrectness/ Accuracy/ Completeness/ Comprehensiveness	Flesch Reading Score	Quality	Rank I Categorization (Diagnosis, Treatment, QA, Others)	Metrics/ Separate Report for CDM	Question Sources
How AI Responds to Common Lung Cancer Questions: ChatGPT vs Google Bard.			**82.5% (120)**<br>\text{Study 1 Accuracy} = \frac{85 \times 100 + 14 \times 75 + 21 \times 50}{120} \approx 87.9\%<br>Likert scale (1-4: 1 is best and 4 is worst): score 1: 85 questions, score 2: 14 questions, score 3: 21 questions<br><br>75.6%-89.3%		70.8% correct (85), 11.7% partially correct (14), 17.5% incorrect (21)			basic knowledge (25)	correct, partially correct, incorrect, and unanswered questions	Lung Imaging Reporting and Data System (Lung-RADS) version 2022 from the American College of Radiology and the Fleischner Society.

Assessing the performance of ChatGPT in answering questions regarding cirrhosis and hepatocellular carcinoma. (Yeo et al., 2023)	**33.3% (9)**<br>- Likert scale (best 1-4 worst): score 1 or 2: 3 questions, score 3: 4 questions; score 4: 2 questions<br><br>1.9%-64.77%		**70.7% (106)**<br>\text{Study 2 Accuracy} = \frac{75 \times 75 + 29 \times 50 + 2 \times 25}{106} \approx 67.5\%<br>Likert scale (best 1-4 worst): score 1 or 2: 75 questions, score 3: 29 questions; score 4: 2 questions<br><br>62.28%-79.22%		- Cirrhosis: 1or 2: >75% for BK (36), Treatment (16), 66.7% diagnosis (3)<br>- HCC: 1or 2: >75% for BK (16), Treatment (30), 3: 50% diagnosis (6), 33.3% (#4)			Basic knowledge (36), treatment (16), lifestyle (22)	1. Comprehensive, 2. Correct but inadequate, 3. Mixed with correct and incorrect/outdated data, and 4. Completely incorrect.	frequently asked questions with cirrhosis or HCC posted by well-regarded professional societies and institutions
Let's chat about cervical cancer: Assessing the accuracy of ChatGPT responses to cervical cancer questions. (Hermann et al. 2023)	**33.3% (3)**<br>Likert scale (best 1-4 worst): score 1 or 2: 1 questions, score 3/4: 2 questions<br><br>20.4%-71.3%	**71.4% (21)**<br>Likert scale (best 1-4 worst): score 1 or 2: 15 questions, score 3/4: 6 questions<br>52.4%-90.4%	**82.8% (64)**<br>\text{Study 3 Accuracy} = \frac{16 \times 75 + 8 \times 37.5}{24} \approx 62.5\%<br>Likert scale (best 1-4 worst): score 1 or 2: 16 questions, score 3/4: 8 questions<br><br>48.1%-85.2%		- Diagnosis: 1/2 score: 33% (1/3), 3/4 score: 67 (2/3)<br>- Treatment: 1/2: 71.4% (15/21), 28.6% (6/21)			Prevention (24), Treatment (21)	1) correct and comprehensive, 2) correct but not comprehensive, 3) some correct, some incorrect, and 4) completely incorrect	professional society websites and the authors' clinical experiences
Is ChatGPT accurate and reliable in answering questions regarding head and neck cancer? Kuşcu et al. (2023)	**98.1% (27)**<br>Likert scale (best 1-4 worst): score 1: 25 questions, score 2: 2 questions<br><br>82.7%-100%	**88.9% (27)**<br>Likert scale (best 1-4 worst): score 1: 24 questions, score 2: 2 questions, score 3: 1 question<br><br>77%-97%	**86.7% (90)**<br>\text{Study 4 Accuracy} = \frac{78 \times 100 + 10 \times 75 + 2 \times 50}{90} \approx 94.4\%<br>Likert scale (best 1-4 worst): score 1: 78 questions, score 2: 10 questions, score 3: 2 question<br><br>79.7-93.5%		- BK: 1: 80.6% (29/36), 2: 16.7% (6/36), 3: 2.8% (1/36)<br>- Diagnosis: 1: 92.6% (25/27), 2: 7.4% (2/27)<br>- Treatment: 1: 88.9% (24/27), 7.4% (2/27), 3.7% (1/27)			Basic knowledge (36), diagnosis (27), treatment (27), Recovery - operative risks – complications – follow-up (n=40)	(1) comprehensive/correct, (2) incomplete/partially correct, (3) a mix of accurate and inaccurate/misleading, and (4) completely inaccurate/irrelevant.	professional societies, institutions, patient support groups, and social media.
Physician Assessment of ChatGPT and Bing Answers to American Cancer Society's Questions to Ask About Your Cancer. Janopaul-Naylor et al. (2024)		**77.6% (117)**<br>Likert scale 1-5 (1 is worst and 5 is best): 3.88 +- 0.38<br><br>70.3-85.3%	**77.6% (117)**<br>\text{Study 5 Accuracy} = \frac{3.87}{5} \times 100 \approx 77.4\%<br>Likert scale 1-5 (1 is worst and 5 is best): 3.87 +- 0.47<br><br>70.3-85.3%		- DISCERN (117 Qs): 4.13 +- 1.31<br>- Score: 3.87 +- 0.47 (Treatment only: 3.88 +- 0.38)			Cancer type (basic knowledge), type of treatment	- DISCERN scale<br>- 5–minimal shortcomings, 3–potentially important but not serious shortcomings, and 1–serious or extensive shortcomings	American Cancer Society's handout of "Questions to Ask About Your Cancer."

ChatGPT in glioma adjuvant therapy decision making: ready to assume the role of a doctor in the tumour board? Haemmerli et al. (2023)	**30%(10)**<br>- Likert scale (worst in agreement 1-10 best in agreement with output from LLM): 3 for 10 cases/questions<br><br>1.6-58.4%	**70% (10)**<br>Likert scale (worst in agreement 1-10 best in agreement with output from LLM): 7 (IQR: 6-8) for 10 cases/questions<br>41.6-98.4%	**50% (10)**<br>\text{Study 6 Accuracy} = \frac{5}{10} \times 100 \approx 50\%<br>Likert scale (worst in agreement 1-10 best in agreement with output from LLM): 5 (IQR: 3-5) for 10 cases/questions<br><br>19-81%		- Diagnosis: 3 (IQR: 1-7.8)<br>- Treatment rec: 7 (IQR 6-8)<br>- Overall: 5 (IQR 3-5)			diagnosis, treatment recommendation, therapy regimen, functional status consideration	'0' indicated complete disagreement, '10' indicated complete agreement and '5' a neutral answer ('neither agreement nor disagreement')	
Answering head and neck cancer questions: An assessment of ChatGPT responses. Wei ei al., 2024			**59.1% (49)**<br>Likert scale (worst 1-5 best): score 4/5: 29 questions, score 2/3: 17 questions, score 1: 4 questions<br><br>44.3%-71.7%	- reading ease: 33.1 ± 10.8<br>- grade level: 14.3 ± 2.4<br>- college level and difficult to read: >30	4/5: 59% (29/49), 1: 8.2% (4/49)	- reading ease: 33.1 ± 10.8<br>- grade level: 14.3 ± 2.4<br>- college level and difficult to read: >30	EQIP: 11.7 ± 2.4	General Information about Head and Neck Cancer: 16 questions<br>Prognosis and Survival: 21 questions<br><br>(prognosis, best hospitals, ICD-10 codes, diagnosis, symptoms, treatment, staging, and cancer awareness)	- Grading was based on the accuracy and completeness of the content(1) very poor, (2) poor, (3) acceptable, (4) good, and (5) very good<br>- Flesch reading scores<br>- Quality: expanded Ensuring Quality Information for Patients (EQIP)	the series of "People Also Ask" (PAA) question prompts using SearchResponse.io
Evaluating ChatGPT Responses on Thyroid Nodules for Patient Education. Campbell et al. (2024)			**69.1%(120)**<br>\text{Study 8 Accuracy} = \frac{3 \times 100 + 34 \times 50 + 83 \times 0}{120} \approx 14.2\%<br>Likert scale (worst 1-3 best): score 1: 3 questions, score 2: 34 questions, score 3: 83 questions<br><br>60.9%-77.3%	Mean FK grade level by prompt type was 14.97 – 2.01 (no prompting), 14.05 – 2.77 (patient-friendly prompting), 13.43 – 2.86 (8th-grade level prompting), and 16.43 – 2.05 (prompting for references).	3/120 (2.5%) incorrect, 34 (28.3%) partially correct, 57 (47.5%) correct, and<br>26 (21.7%) correct with reference ("correct+")	Mean FK grade level by prompt type was 14.97 – 2.01 (no prompting), 14.05 – 2.77 (patient-friendly prompting), 13.43 – 2.86 (8th-grade level prompting), and 16.43 – 2.05 (prompting for references).		epidemiology (1–5), diagnosis (6–15), prognosis (16–20), and management (21–30).	- medical accuracy and clinical appropriateness using a four-part hierarchical scale: incorrect, partially correct, correct, or correct with a reference ("correct+")<br>- FK reading score	

Evaluating AI in medicine: a comparative analysis of expert and ChatGPT responses to colorectal cancer questions. Peng et al. (2024)			**92%(131)**<br>0-1 scale (1 is best): 0.92 for 131 questions<br><br>87.9-96.9%		- 0-1 (131 Qs): Comprehensive (0.85), accuracy (0.97), final score (0.92)			basic information (14), surgical management (16), internal medicine treatments (34), radiation therapy (7), interventional treatments (8), ostomy care (17), deep vein care (14),<br>pain control (21)	- comprehensiveness, accuracy, final standardized score (1-10)	Colorectal Cancer: Your Questions Answered
Urological Cancers and ChatGPT: Assessing the Quality of Information and Possible Risks for Patients. Ozgor et al. (2024)			**85.9%(78)**<br>- prostate cancer (24): 2: 1 (4.2%), 3: 2 (8.3%), 4: 2 (8.3%), 5: 17 (70.8%)<br>- bladder cancer (24): 3: 2 (8.3%), 4: 6 (25%), 5: 16 (66.7%)<br>- kidney cancer (18): 3: 3(16.7%), 4: 3(16.7%), 5: 12(66.7%)<br>- testicular cancer (12): 3: 1(8.3%), 4: 1(8.3%), 5: 10(83.3%)<br><br>78.2%-93.5%				Diagnosis & treatment:<br>- prostate cancer (24): 2: 1 (4.2%), 3: 2 (8.3%), 4: 2 (8.3%), 5: 17 (70.8%)<br>- bladder cancer (24): 3: 2 (8.3%), 4: 6 (25%), 5: 16 (66.7%)<br>- kidney cancer (18): 3: 3(16.7%), 4: 3(16.7%), 5: 12(66.7%)<br>- testicular cancer (12): 3: 1(8.3%), 4: 1(8.3%), 5: 10(83.3%)	Prostate cancer: symptom 21, diagnosis/treatment 24, prognosis/follow up 20<br>Bladder cancer: symptom 18, diagnosis/treatment 24, prognosis/follow up 20<br>Kidney cancer: symptom 16, diagnosis/treatment 18, prognosis/follow up 13<br>Testicular cancer: symptom 14, diagnosis/treatment 12, prognosis/follow up 10	- GQS: 1-5 1. Poor quality 5. Excellent quality	
Conformity of ChatGPT recommendations with the AUA/SUFU guideline on postprostatectomy urinary incontinence. Pinto et al. (2024)			**98%(20)**<br>correct: 19/20, incorrect: 1/20<br><br>85%-100%		- total score: 18/20<br>- Concep Qs: correct: 8/10, partially correct: 2/10<br>- case-based: correct: 9/10, incorrect: 1/10			Treatment: preprostate treatment (two questions), postprostate treatment<br>(two questions), evaluation of incontinence after prostate<br>treatment (three questions) and treatment options (three questions).	- correct (1 point); partially correct (0.5 point), or incorrect (0 point)	The Incontinence After Prostate Treatment: AUA/SUFU Guideline.

Artificial intelligence large language model ChatGPT: is it a trustworthy and reliable source of information for sarcoma patients? Valentini et al. (2024)		**45%(25)**<br>Likert scale 1-5 (1 is best and 5 is worst): score 1: 3/11 (27%), Score 2: 2/11 (18%), score 3: 3/11 (27%). score 4: 3/11 (27%)<br><br>15.8-75.1%	**72% (25)**<br>accuracy: 3.7 points; IQR, 2.5–4.2 points; completeness: 3.5 points; IQR, 2.8–4.0<br><br>54-80.6%		evaluation metric of how appropriate the response was for patients (median, 3.7 points; IQR, 2.5–4.2 points), which were significantly higher compared to the accuracy scores (median, 3.3 points; IQR, 2.0–4.2 points; p =0.035). On the other hand, with the numbers we had the differences between the accuracy and completeness scores (median, 3.5 points; IQR, 2.8–4.0; p =0.066)		total score 18.3 (12.3-20.3)<br>- Six of the 25 responses (24%) were classified as very good, 9/25 (36%) as good, while 5/25 answers each (20%) were defined as poor and very poor<br>- ChatGPT fared best with general inquiries, achieving good and very good overall scores in 3/9 (33%) and 4/9 (44%) questions, respectively. Only 1/9 (11%) response each was rated as poor and very poor, respectively (Figure 3). On the other hand, the bot fared considerably worse on treatment-related questions, achieving good and very good overall scores in 3/11 (27%) and 2/11 (18%), respectively. 3/11 (27%) responses each were classified as poor and very poor, respectively	25 sarcoma-related questions (5 definitions, 9 general questions, and 11 treatment-related inquiries)	- 5 different metrics using a 5-point Likert scale: completeness, misleadingness, accuracy, being up-to-date, and appropriateness	

Comparison of Large Language Models in Answering Immuno-Oncology Questions: A Cross-Sectional Study. Iannantuono et al. (2024)			**68.1% (60)** for 60 questions<br><br>56.2%-80%		- Reproducibility (57%)<br>- Accuracy (43 (75.4))<br>- relevancy (41(71.9%))	- highly readable answers (57%)		General info (basic knowledge)	- Answer returned (T/F)<br>- Reproducibility (T/F)<br>- Accuracy (1-3)<br>- Readability (1-3)<br>- Relevance (1-3)	
Quality of Large Language Model Responses to Radiation Oncology Patient Care Questions. Yalamanchili et al. (2024)		**87.3%(41)**<br>41 treatment modality-specific Qs: 91% of responses for relative factual correctness, 80% of responses for relative completeness, and 91% of responses for relative conciseness<br><br>77.8-97.8%	**87.3% (115)**<br>108 responses (94%) in relative correctness, 89 responses (77%) in completeness,<br>and 105 responses (91%) in conciseness<br><br>80.8-93.1%		- 115 professional websites: 108 responses (94%) in relative correctness, 89 responses (77%) in completeness,<br>and 105 responses (91%) in conciseness<br>- 45 general Qs: 100% for factual correctness, 90% for relative completeness, and 83% for relative conciseness<br>- 41 treatment modality-specific Qs: 91% of responses for relative factual correctness, 80% of responses for relative completeness, and 91% of responses<br>for relative conciseness<br>- site-specific: 92% of responses for relative factual correctness, 66% of responses for relative completeness,<br>and 98% of responses for relative conciseness			29 general radiation oncology questions from Cancer.gov; 45 treatment modality–specific questions and 41 cancer subsite–specific questions	- relative correctness, conciseness, completeness, and potential harm compared with online expert answers on a 5-point Likert scale (1: "much worse," 2: "somewhat worse," 3: "the same," 4: "somewhat better," and 5: "much better")<br>- 10 major readability assessment scales	websites (accessed February 1 to March 20, 2023) affiliated with the National Cancer Institute and the Radiological Society of North America.
Chat-GPT on brain tumors: An examination of Artificial Intelligence/Machine Learning's ability to provide diagnoses and treatment plans for example neuro-oncology cases. Kozel et al. (2024)	**85% (20)** for diagnosis for 20 cases/questions<br><br>69.3-100%		**80%(20)**<br>85% overall accuracy for diagnosis and 75% overall accuracy for treatment plan for 20 cases<br><br>62.4%-97.5%		- 85% overall accuracy for diagnosis, with a 92.3% for benign tumors and a 71.4% accuracy for malignant tumors, and a 75% overall accuracy for treatment plan, with a 84.6% accuracy for benign tumors and a 57.1% accuracy for malignant tumors		- ChatGPT-4 received an average score of 8.3 for its diagnoses of nineteen of the twenty tumors, 8.4 for its treatment options, and 8.5 for its treatment plans	Diagnosis, treatment options and plans for 20 cases	Y<br>- accuracy (Y/N)<br>- response grading (1-10)	

Assessing the role of GPT-4 in thyroid ultrasound diagnosis and treatment recommendations: enhancing interpretability with a chain of thought approach. Wang et al. (2024)	**73.6% (109)**<br>- 3.68/5 (Likert 1-5, 1 worst, 5 best) (95% confidence interval (CI): 3.52–3.8) for 109 cases<br><br>68-84%		**76%(109)**<br>3.80/5 (Likert 1-5, 1 worst, 5 best) (95% confidence interval (CI): 3.52–3.8) for 109 cases<br><br>68-84%		- Diagnosis accuracy (3.68/5 95% confidence interval (CI): 3.52–3.8)<br>- general evaluation, GPT-4's mean score was 3.80 (95% CI: 3.67–3.94)	- clarity of expression, it achieved a mean score of 4.26 (95% CI: 4.16–4.37)		Diagnosis and treatment recommendation for cases	- diagnostic accuracy<br>- Turing test for human authorship (1-5)<br>- reproducibility<br>- evaluation (positive (praises AI-generated report for accuracy, clarity, or usefulness), negative (highlights concerns, such as inaccuracies, ambiguities, or irrelevance),<br>or neutral (neither praises nor criticizes; offers general observations or suggestions))	
A chat about actinic keratosis: Examining capabilities and user experience of ChatGPT as a digital health technology in dermato-oncology Lent et al. (2024)	**37.5% (8)** for 3 out of 8 questions<br><br>3.4-71%	3 out of 11 are accurate (**27.3% (11)**)<br><br>5.1-53.7%	18/38 questions(**47.4% (38)**) accurate<br><br>31.4-63.2%		- 18/38 questions(47.4%) accurately in real‑time; but only 12 questions(31.6%) were answered with an accurate, current andcomplete response<br>- patient education, with 11 of 19 responses ratedaccurate (57.9%) and 9 of which were also current andcomplete (47.4%). In the category of diagnosis, three outof eight responses were rated accurate (37.5%) as well ascurrent and complete. Questions pertaining to treatmentreceived the lowest accuracy rate with only 3 out of 11responses considered factually accurate (27.3%), none ofwhich were rated current and complete.			38 Qs about patient education, diagnosis, and treatment	- actual accuracy,currency of information, and completeness (Y/N)	

Current Strengths and Weaknesses of ChatGPT as a Resource for Radiation Oncology Patients and Providers. Floyd et al. (2024)			**65.8%(252)**<br>Likert scale (worst 1-3 best): score 1: 86 questions, score 2: 66 questions, score 3: 100 questions<br><br>60-71%		- A total of 86 (34.1%) answers were found to contain inaccurate information, 66 (26.2%) contained correct information but were found to be missing essential context, and 100 (39.7%) responses were graded as correct and comprehensive<br>- landmark study Qs: Of the 40 ChatGPT3.5 responses to questions related to these seminal studies, a total of 22 (55.0%) answers were found to contain inaccurate information, 4 (10.0%) contained correct information but were found to be missing essential context, and 14 (35.0%) responses to questions were graded as correct and comprehensive			Basic knowledge for 28 patient-centered Qs (diagnosis, treatment, management, survivorship)	- 0 if there was any incorrect information, 1 if any essential content was missing, or 2 if the response was correct and appropriately comprehensive for the length of the response<br>- DICERN score	
Evaluating large language models on a highly-specialized topic, radiation oncology physics Holmes et al. (2023)			**75% (100)** accuracy for 100 questions<br><br>66.5-83.5%		- GPT-4 75% accuracy for all Qs (SD 1.8%) (basic physics 92%, teartment planning 69%, adv. TP 89%)<br>- GPT-4 had 67% of questions correct in each trial, however it also showed a propensity for confusion, getting 14% of questions incorrect in each trial.			basic physics (12 questions), radiation measurements (10 questions), treatment planning (20 questions), imaging modalities and applications in radiotherapy (17 questions), brachytherapy (13 questions), advanced treatment planning and special procedures (16 questions), and safety, quality assurance (QA), and radiation protection (12 questions).	compare LLM with human graders<br>- accuracy (Y/N)<br>- consistency: SD, average correlation<br>- confidence: # of correct answers<br>- deductive reasoning/vote...	
